# Wormian Bone in the Anterior Fontanelle of an Otherwise Well Neonate

**DOI:** 10.7759/cureus.4741

**Published:** 2019-05-23

**Authors:** Arthur T Pickett, M. Alisha Montes

**Affiliations:** 1 Internal Medicine, Memorial University of Newfoundland, St. John's, CAN; 2 Pediatrics, Memorial University of Newfoundland, St. John's, CAN

**Keywords:** anterior fontanelle, neonate, wormian bone, skull

## Abstract

Wormian bones are supernumerary bones of the skull that form as a result of extra-ossification centers during development in utero. They are a common finding that typically lie within and are surrounded by suture lines, especially the lambdoidal and coronal sutures. Wormian bones located within the fontanelles of neonates have a lower reported incidence and have been associated with various disorders such as craniosynostosis and osteogenesis imperfecta. Few reports have documented the existence of a Wormian bone in the fontanelle of an infant without complications or underlying pathology. There are limited resources or case reports that discuss a Wormian bone as a variant of normal.

This report details the case of a child with an isolated Wormian bone within the anterior fontanelle discovered before discharge from hospital at birth. The child went on to have normal growth of his head circumference and did not have any further developmental complications after follow-up.

## Introduction

The bones comprising the skull of a developing human do not fuse until after birth. Wormian bones form as a result of extra ossification centers in utero. These bones, named in honour of the Dutch anatomist Olaus Wormius by Thomas Bartholin, are commonly found in the pediatric population with reports stating as much as 53% of children having radiographic evidence of one or more [1]. These supernumerary bones of the pediatric skull are often identified within suture lines, most notably within and surrounded by the lambdoidal or coronal sutures. Much lower in incidence are those located within the fontanelles of a developing skull. Their formation is thought to be associated with tension forces being applied to the skull causing variations within the normal development of bone along suture lines [2].

Wormian bones may be associated with a variety of disorders such as craniosynostosis or osteogenesis imperfecta. This, however, is based on multiple characteristics including size, shape, location, and the number of supernumerary bones. For example, osteogenesis imperfecta has been associated with children having 10 or more Wormian bones [3].

Cases of Wormian bones within the anterior fontanelle requiring medical or surgical intervention have been previously reported [4]. In addition, there have been reports of anterior fontanelle Wormian bones with other associated clinical findings such as exophthalmos or vascular malformations [5-6]. One small clinical study discussed the possibility of normal development in some children with the anterior fontanelle Wormian bone, but with many of the subjects requiring surgical intervention for craniosynostosis [7]. Parents of such children are left with minimal resources for the explanation of the finding without its relation to serious medical sequelae. Therefore, this case report highlights a case of a child with the incidental finding of an anterior fontanelle Wormian bone with normal development and no medical sequelae or surgical intervention required. 

## Case presentation

History

The child, in this case, was born to a 33-year-old G2P1A1 (Gravida 2, Para 1, Abortus 1) mother. Her serology was protective and blood group was O negative. Her past medical history was significant for congenital heart disease, gestational hypertension, and infertility requiring in vitro fertilization for both pregnancies. She was not on any medications during pregnancy and denied consumption of alcohol, drugs or cigarettes. She had an anatomy ultrasound and fetal echocardiography at 20 weeks which were both normal. Group B Streptococcal status was unknown. The child was born at a gestational age of 38 weeks and 4 days by repeat cesarean section. The Apgar scores were nine at both one and five minutes. There was no resuscitation required. On the World Health Organization (WHO) growth charts, birth-weight was 3710 grams (75th centile), head circumference was 35.5 cm (75th centile), and height was 51 cm (75th centile).

Physical exam

On general inspection, the child looked alert and showed no signs of distress. There were no obvious dysmorphic features. The anterior fontanelle was hard on palpation and there was no obvious opening; however, the presumed ossification was mobile suggesting no premature fusion. The posterior fontanelle was not palpated. The shape of the skull was normal without signs of abnormalities such as molding or cephalohematoma, and there was no ridging of the cranial sutures. The rest of the musculoskeletal exam was normal. Neurological exam revealed normal grade 0 tone, 1+ reflexes, and symmetrical movements. Pupils were equal and reactive. Red reflex was symmetrical. A cardiovascular exam revealed normal heart sounds with no murmurs or added sounds. Femoral and brachial pulses were palpable and capillary refill was normal. A respiratory exam revealed good air entry bilaterally with no adventitious sounds. Nasal passages were patent. Examination of the oropharynx was normal with no obvious cleft. Gag reflex was present and there was no tongue tie. The abdomen was soft and non-tender with no hepatosplenomegaly or hernias. Genitalia was normal with both testes descended. Examination of the hips and spine was normal. Examination of the skin and the sclera was normal.

Investigations

**Figure 1:**
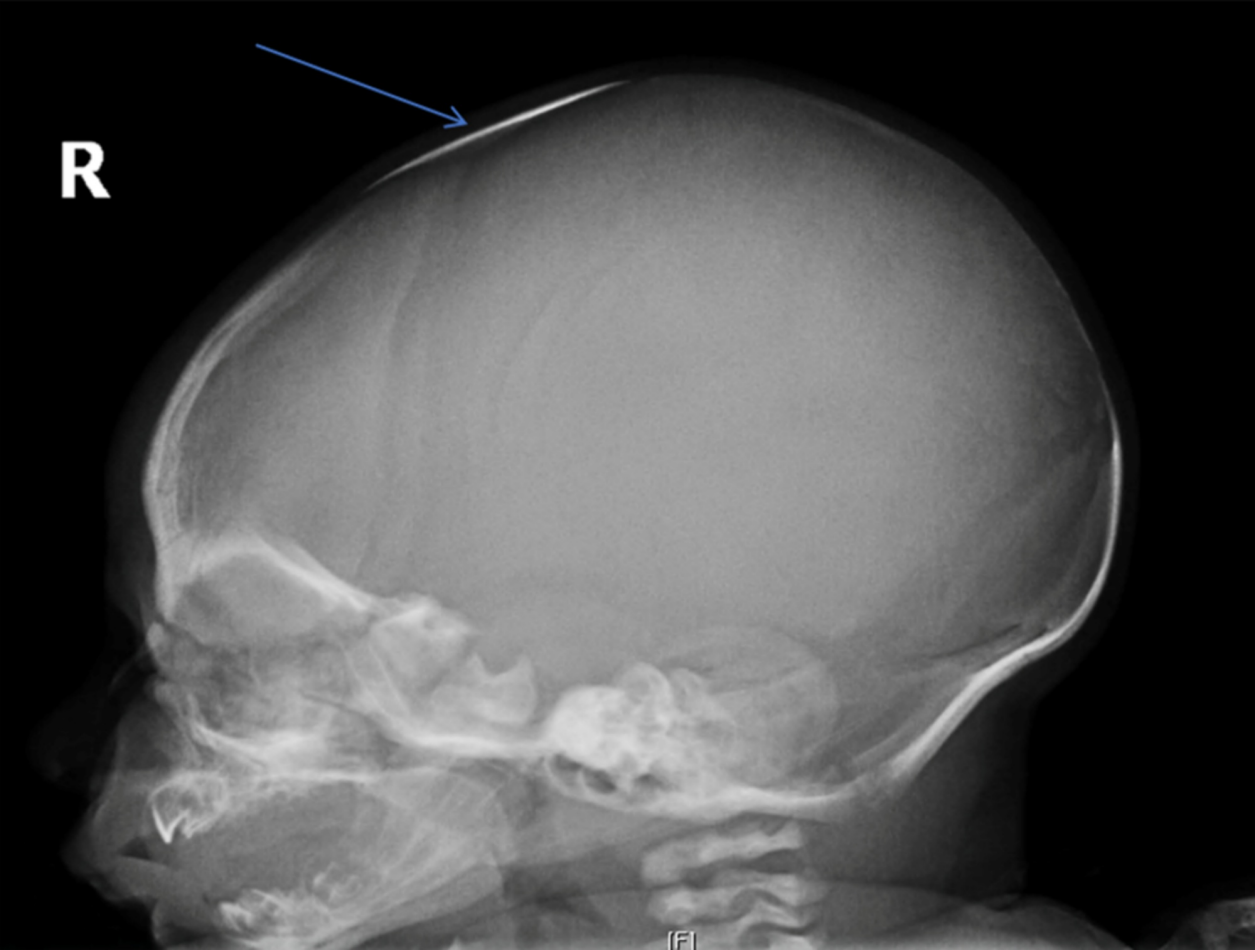
Left lateral view from the cranial X-ray series Blue arrow depicts the presence of Wormian bone in the anterior fontanelle.

**Figure 2:**
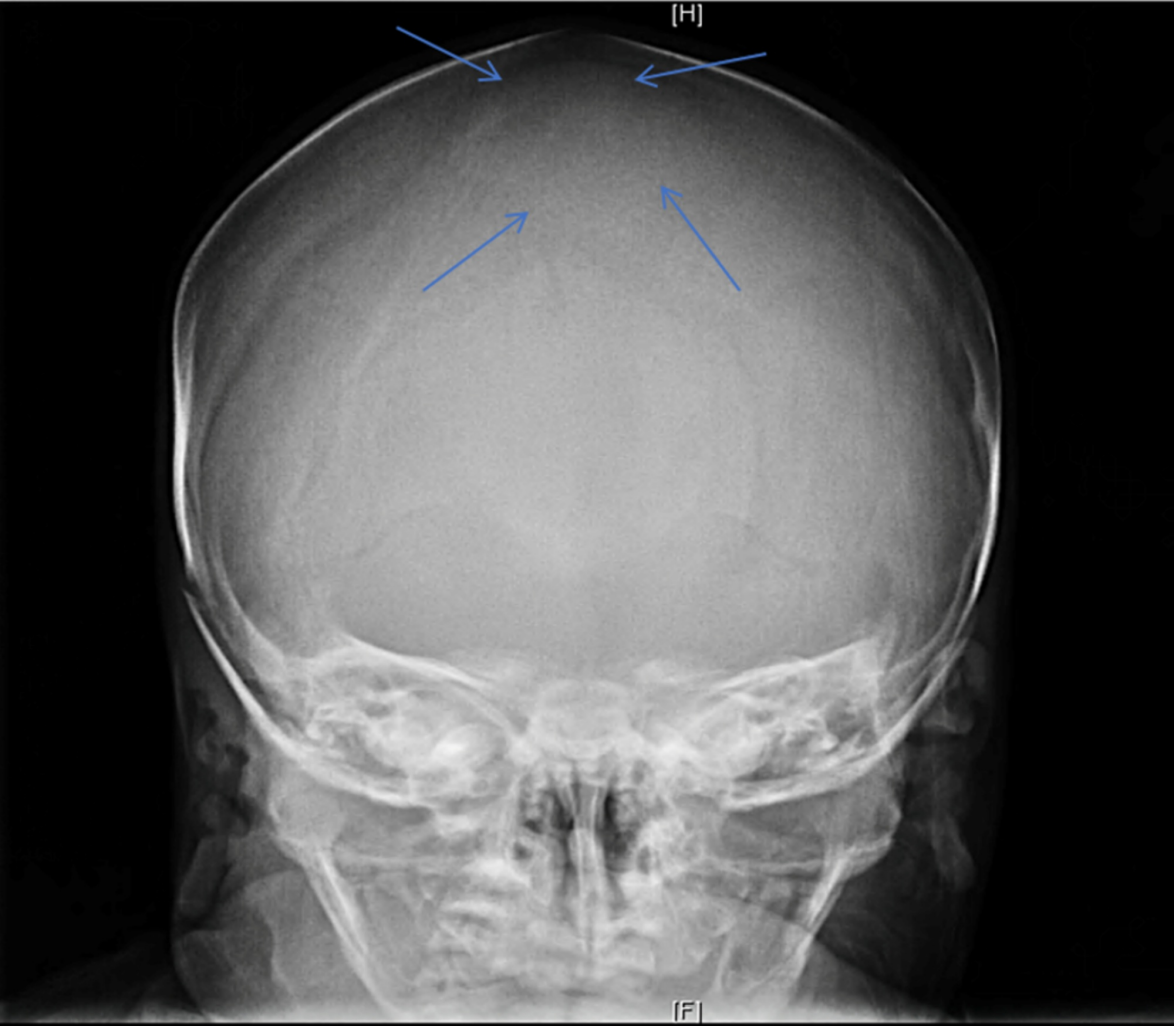
Anterior view in the cranial X-ray series Blue arrows depict the outline of Wormian bone in the anterior fontanelle.

Cranial X-ray series was ordered after the discovery of the bony structure within the anterior fontanelle. The X-ray showed a large bone in the location of the anterior fontanelle compatible with a Wormian bone (Figure 1, 2). There was no evidence of craniosynostosis as all the cranial sutures were patent.

Outcome

Neurosurgery was consulted during admission on the newborn floor. The diagnosis of a Wormian bone was confirmed and the team recommended close follow up of the growth of the head circumference. No surgical management was recommended as there was no evidence of craniosynostosis.

Genetics was also consulted during admission to ascertain whether genetic testing was required. The genetics team did an evaluation and did not find any indications that the Wormian bone was part of an overarching genetic syndrome and did not recommend genetic testing.

Follow-up with a well newborn clinic was arranged and the child was seen at four weeks, 2.5 months, and four months. At each appointment, the child’s head circumference was measured. At 4 weeks, it was 38.4 cm; at 2.5 months, it was 41.3 cm, and at four months, it was 43cm. When plotted on the WHO growth charts, the head circumference was growing symmetrically along the 85th centile. The child’s weight and height were also growing symmetrically along the 50th centile. The child was exclusively breastfed and had no issues with feeding. He was developing appropriately and did not have any signs of developmental regression or neurological abnormalities. There were no signs of fractures in any of the other bones. His examinations were completely normal through all follow-up appointments. He did not demonstrate any signs of increased intracranial pressure and his bowel and bladder functions were normal.

## Discussion

Wormian bones are a normal anatomical variant of the skull, which often lie within the suture lines. Hence, they generally go unnoticed [1]. Less common is a Wormian bone within the anterior fontanelle of a newborn child. The fontanelles are important for normal growth of a child’s head. Most concerning with a Wormian bone within the anterior fontanelle is craniosynostosis. If this were to occur, surgical resection of the fused suture lines would be indicated to ensure proper growth of head circumference [7]. Wormian bones have been shown to be associated with genetic conditions such as osteogenesis imperfecta, cleidocranial dysplasia, and hypophosphatasia, however they are not pathognomonic [3].

No case reports in the literature discuss Wormian bones within the anterior fontanelle as a normal variant. Therefore, this case aims to provide information to parents and caregivers about the possibility of a Wormian bone in the anterior fontanelle as a normal variant that requires no intervention or further testing.

The child in this study was born via repeat Caesarean section following a pregnancy with no complications. The child did well throughout its stay on the neonatal ward. All follow up newborn care appointments were normal, most notably with normal growth of head circumference. Careful attention was given to neurodevelopment and signs or symptoms associated with increased intracranial pressure. Bony abnormalities that could be suggestive of osteogenesis imperfecta such as pathologic fractures were also absent.

Although the risk of complication for craniosynostosis or association with other diseases may be increased, a thorough work-up and proper follow up can allow physicians to accurately inform parents that an anterior fontanelle Wormian bone can be a normal variant. Healthcare professionals may reassure patients of normalcy in the absence of other signs or symptoms including increased intracranial pressure, micro or macrocephaly, ridging of the cranial sutures, pathologic fractures, dysmorphic features such as exophthalmos and other facial deformities, or concurrent vascular malformations [5-6].

## Conclusions

This case presented a neonate with a Wormian bone in the anterior fontanelle, without medical sequalae during follow up appointments. Providers may reassure parents of these children that such a finding may be a normal variant in the absence of other comorbid signs or symptoms.
